# Prophylactic Resorbable Synthetic Mesh to Prevent Wound Dehiscence and Incisional Hernia in High High-risk Laparotomy: A Pilot Study of Using TIGR Matrix Mesh

**DOI:** 10.3389/fsurg.2016.00028

**Published:** 2016-05-18

**Authors:** Harald Söderbäck, Haile Mahteme, Per Hellman, Gabriel Sandblom

**Affiliations:** ^1^Department of Surgery, Capio St Göran, Stockholm, Sweden; ^2^Institution for Surgical Sciences, Uppsala University, Uppsala, Sweden; ^3^Department of Surgery, Uppsala University Hospital, Uppsala, Sweden; ^4^Karolinska Institutet, CLINTEC, Stockholm, Sweden

**Keywords:** wound dehiscence, incisional hernia, prevention, mesh, synthetic, resorbable

## Abstract

**Background:**

Wound dehiscence and incisional hernia are potentially serious complications following abdominal surgery, especially if performed through a midline incision. Although prophylactic reinforcement with on-lay mesh has been shown to reduce this risk, a permanent mesh carries the risk of seroma formation, infection, and persistent pain. The aim of this study was to assess the safety of a reabsorbable on-lay mesh to reinforce the midline suture in patients with high risk for wound dehiscence or incisional hernia.

**Method:**

Sixteen patients with three or more risk factors for wound dehiscence or incisional hernia were included. A TIGR^®^ Matrix mesh, composed of a mixture of 40% copolymer fibers of polyglycolide, polylactide, and polytrimethylene carbonate and 60% copolymer fibers of polylactide and polytrimethylene carbonate, was placed on the aponeurosis with an overlap of five on either side and fixated with continuous monofilament polydioxanone suture. All postoperative complications were registered at clinical follow-up.

**Results:**

Mean follow-up was 9 months. One patient developed a seroma that needed drainage and antibiotic treatment. One patient had a wound infection that needed antibiotic treatment. There was no complication requiring a reoperation. No wound dehiscence or incisional hernia was seen.

**Conclusion:**

On-lay placement of TIGR^®^ Matrix is safe and may provide a feasible way of reinforcing the suture line in patients with high risk for postoperative wound dehiscence or incisional hernia. Larger samples are required, however, if one is to draw any conclusion regarding the safety and effectiveness of this technique.

## Introduction

Wound dehiscence and incisional hernia after laparotomy are serious and costly complications. The problem of incisional hernia is often underestimated since the hernia may not be clinically obvious until several years after the operation and thus does not always come to the attention of the surgeon. Recent studies have shown that the risk for wound dehiscence and incisional hernia can be reduced by meticulous suture technique (1). However, even with a careful suturing around 5% of patients undergoing laparotomy suffer from wound complications (2) that may lead to prolonged hospital stay and severe morbidity (3). Risk factors for wound dehiscence and incisional hernia include acute surgery, reoperation, age over 80 years, generalized malignant disease, chronic obstructive pulmonary disease (COPD), hypoalbuminemia, sepsis, obesity, anemia, insulin-treated diabetes with secondary complications, steroid treatment, smoking, chemotherapy, radiation therapy of the abdominal wall, and resection of the abdominal wall (3–8).

Reinforcement of the suture line with a mesh may be an effective way of preventing wound dehiscence (9). By placing a polypropylene mesh on the aponeurosis after closure of the midline, the risk for incisional hernia has been shown to be reduced by at least two-thirds (10, 11). A synthetic mesh left permanently in the subcutaneous tissue, however, is associated with wound complications that may be difficult to manage, including infection, seroma, and chronic pain (12).

A possible way of reducing the frequency of wound complications due to permanent mesh placement may be to use a slowly resorbable mesh. Biological meshes have been used for strengthening of the abdominal wall in cases where a gradually resolving mesh is necessary for the long-term outcome. To date, however, biological meshes have been very expensive and do not always have optimal properties for this purpose. The TIGR^®^ Matrix Surgical Mesh, on the other hand, is a completely synthetic mesh that is gradually absorbed. It is intended for use in reinforcement of soft tissues where weakness exists, in procedures involving repair of hernia and abdominal wall defect, abdominal wall reinforcement, and muscle flap reinforcement (13).

The aim of this pilot study was to see if TIGR Matrix is feasible as a mesh for prophylactic reinforcement in high-risk incisions.

## Materials and Methods

This study included patients from Uppsala University Hospital, the Uppsala Cancer Clinic, and Karolinska University Hospital, Huddinge, undergoing surgery through a midline incision. Inclusion criteria were the presence of at least three documented risk factors for incisional hernia or wound dehiscence.

Listed risk factors for this study were: reoperation, age over 80 years, generalized malignant decease (presence of distant metastases at the time of surgery), COPD Grades III–IV according to the GOLD classification (FEV1 <50% of the expected), serum albumin level <20 g/l, sepsis, infection in combination with two or more of the following: abnormal body temperature, heart rate, respiratory rate or blood gas, and white blood cell count, BMI >35, hemoglobin <80 g/l, diabetes with secondary complications (angiopathy, nephropathy, or neuropathy) and insulin treatment, steroid treatment (with at least 1 mg betamethasone daily or equivalent) for 7 days preoperatively, smoking (at least 10 cigarettes a day for 1 year), chemotherapy (last administration within 2 weeks prior to surgery), and radiation therapy of the abdominal wall.

The abdominal wall was closed with continuous Polydioxanon Suture (PDS) with a 4:1 ratio of the suture to incision length. After closing the midline incision according to usual routines, the aponeurosis was reinforced by on-lay TIGR^®^ Matrix Surgical Mesh (Novus Scientific Uppsala Sweden).

TIGR^®^ Matrix Surgical Mesh is composed of two different synthetic resorbable fibers having different degradation characteristics. The first fiber, constituting 40% of the matrix, is a copolymer of polyglycolide, polylactide, and polytrimethylene carbonate. The second fiber, making up 60% of the matrix, is a copolymer of polylactide and polytrimethylene carbonate. Both fibers are degraded by bulk hydrolysis, resulting in a decreasing tensile strength caused by loss of fibers. *In vitro* tests have shown that the first fiber (polyglycolide, polylactide, and polytrimethylene carbonate) loses its functional tensile strength after 2 weeks, and *in vivo* studies in the abdominal wall of sheep have shown that it is fully absorbed after 4 months (14). Corresponding figures for the second fiber (polylactide and polytrimethylene carbonate) are 9 months and approximately 36 months, respectively. As the first fiber is resorbed, the elasticity increases, which improves collagen formation. The TIGR Matrix Surgical Mesh is a resorbable mesh implant, classified as a Class III device in accordance with the European Medical Device Directive (MDD) 93/42/EEC, Annex IX, Section 2.4, Rule 8.

**Figure 1:**
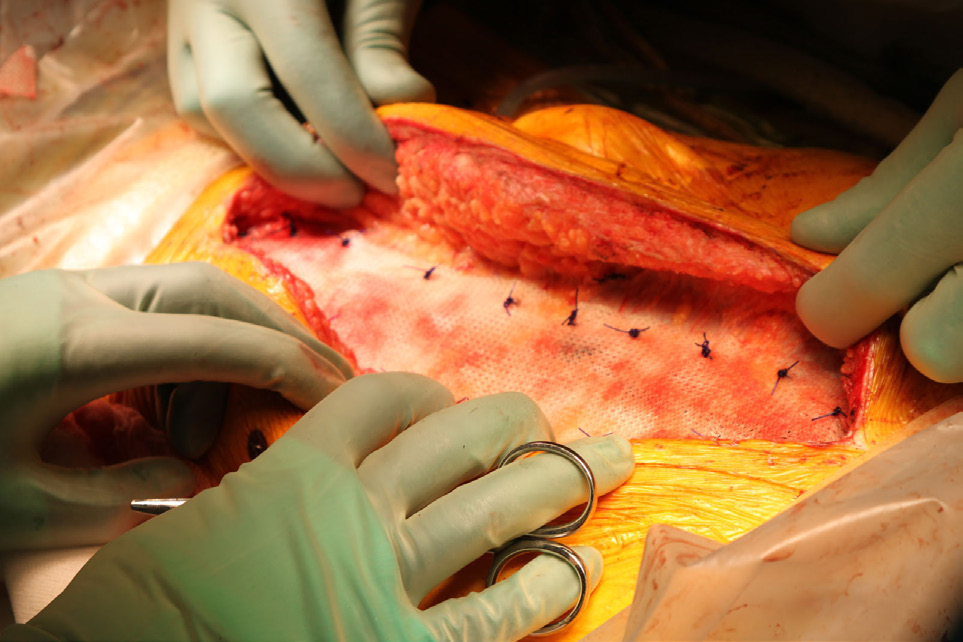
**TIGR^®^ Matrix Surgical Mesh placed onlay to a closed midline incision**.

In the present study, TIGR^®^ Matrix was used to reinforce the midline suture. After closing the midline, with best possible suturing technique, a TIGR^®^ Matrix mesh was placed on-lay on the rectal aponeurosis with an overlap of 5 cm on either side. The mesh was fixated with a continuous PDS 2-0 suture on each side parallel to the midline incision (Figure 1), followed by skin closure. All patients were followed up according to the clinical practice at each unit, but always including a follow-up visit 1 month after surgery. Endpoints in this study were wound dehiscence, wound infection, seroma, and persistent pain.

## Results

Sixteen patients were included in the study. Baseline data and outcome are presented in Table 1. Most of the patients underwent peritonectomy with hyperthermic intraperitoneal chemotherapy (HIPEC) for peritoneal metastases. Mean follow-up time in the study was 9 months. Of the 16 patients, 1 had a seroma that needed drainage and antibiotic treatment, and 1 had a wound infection that needed antibiotic treatment. There was no complication requiring a reoperation. No dehiscence or incisional hernia was seen. None of the surgeons found the on-lay fixation technically difficult or time consuming. There were no major complaints regarding discomfort from the mesh.

**Table 1:** **Study group**.

Patient	Sex	Age (years)	Operating unit	Indication for surgery	Risk factors	Adverse events	Comments
1	M	42	UCC	PM + AWM	E + I + K	–	
2	F	49	UCC	PM	B + E + I + K	–	
3	F	58	UCC	PM	A + B + C + E + I	–	
4	F	65	UCC	PM + AWM	E + I + K	–	
5	F	65	UCC	PM + AWM	E + F + K	–	
6	F	65	UCC	PM + AWM	E + I + K	–	
7	F	63	UCC	PM + AWM	E + I + K	–	
8	F	55	UCC	PM + AWM	E + I + K	–	
9	F	70	UCC	PM + AWM	E + I + K	–	
10	F	67	UCC	PM + AWM	A + D + E + I + K	Seroma	Drain + AB
11	F	45	UCC	PM + AWM	B + E + I + K	–	
12	M	32	UUH	Pancreatic cancer	B + K	–	
13	M	77	Huddinge	Reoperation for dehiscence	G + H + I	–	
14	M	78	Huddinge	Reoperation for dehiscence	G + H + I + J	Superficial wound infection	AB
15	F	69	Huddinge	Reoperation for dehiscence	B + F + I + L	–	
16	M	67	Huddinge	Reoperation for dehiscence	B + D + F	–	

*PM, peritoneal metastases; AWM, abdominal wall metastases; UCC, Uppsala Cancer Clinic; UUH, Uppsala University Hospital; AB, antibiotic treatment*.

*Risk factors: A, COPD; B, BMI >35; C, insulin-treated diabetes; D, smoking; E, ongoing/previous cytostatic treatment: F, laparotomy + GI resection <1 month previously; G, reoperation; H, acute surgery; I, generalized cancer; J, albumin <20 g/l; K, abdominal wall resection; L, steroid treatment*.

## Discussion

On-lay placement of TIGR^®^ Matrix is safe and may be a feasible way of reinforcing the midline suture in patients with high risk for postoperative wound dehiscence or incisional hernia. No major complication, incisional hernia, or dehiscence was seen after follow-up of at least 6 months. However, larger samples are required if one is to draw any conclusion regarding the safety and effectiveness of this technique.

Three surgeons from three different hospitals performed the procedures. None of the surgeons found the procedure technically difficult or time consuming. One patient developed a seroma that caused moderate symptoms. As the mesh is resorbed, the seroma formation should also resolve. Similarly, pain from the mesh as well as non-septic mesh inflammation may also be managed conservatively, awaiting the gradual resorption of the mesh.

The need for relaparotomy is not rare in patients undergoing abdominal surgery. Performing a laparotomy through a mesh from a previous laparotomy may be complicated, with increased risk for infection or other wound complication. This problem is also avoided by using a resorbable mesh.

The present study demonstrates that reinforcement of the midline suture after laparotomy using a synthetic resorbable mesh is not associated with major adverse events. It was too small, however, to be able to provide definite evidence for the effectiveness of the technique. Whether or not late incisional hernias develop when the mesh is completely resorbed can only be determined in studies with longer follow-up and under more controlled circumstances. The present study was intended as a pilot study before the initiation of a larger randomized clinical trial with incisional hernia and wound dehiscence as primary endpoints.

In conclusion, the prophylactic placement of an on-lay slowly resorbable mesh to strengthen the midline suture in patients with high risk for incisional hernia or wound dehiscence appears to be a safe technique. A larger controlled and randomized trial is required to fully evaluate the effect of a prophylactic resorbable mesh on the development of incisional hernia and wound dehiscence.

## Ethics Statement

The study was approved by the Ethical Review Board of Stockholm (2015/440-31).

## Author Contributions

All authors listed have made substantial, direct, and intellectual contribution to the work and approved it for publication.

## Conflict of Interest Statement

HS declares no conflict of interest. HM declares no conflict of interest. PH declares no conflict of interest. GS reports non-financial support from Novus Scientific during the conduct of the study. Novus has supplied with TIGR^®^ Matrix, and the technical information about TIGR^®^ Matrix but has not been involved in collecting or processing data.
